# Dietary Proanthocyanidins Are Associated with Changes in the Fecal Microbiota and Serum Bile Acid Profiles of LPS-Challenged Weaned Piglets

**DOI:** 10.3390/microorganisms14102324

**Published:** 2026-10-12

**Authors:** Xiyue Chang, Aiying Yu, Lian Chen, Dan Wang, Zhiyi Li, Jianan Wu, Weiguo Zhao, Zhenjiang Wang, Sen Lin

**Affiliations:** 1 Key Laboratory of Urban Agriculture in South China, Sericultural & Agri-Food Research Institute, Guangdong Academy of Agricultural Sciences, Guangzhou 510640, China; 2 Jiangsu Key Laboratory of Sericulture Biology and Biotechnology, School of Biotechnology, Jiangsu University of Science and Technology, Zhenjiang 212100, China

**Keywords:** proanthocyanidins, weaned piglets, gut microbiota, bile acids, lipopolysaccharide

## Abstract

Inflammatory stress can disrupt gut microbial communities and bile acid (BA) metabolism in weaned piglets; whether dietary proanthocyanidins (PACs) can alleviate such alterations remains unclear. Eighteen 21-day-old Duroc × Landrace barrows were randomly assigned to control (CON), lipopolysaccharide-challenged (LPS), and LPS-challenged PAC-supplemented (LPS + PAC; 250 mg/kg diet) groups for a 21-day trial. Fecal microbiota and serum BAs were assessed by 16S rRNA gene sequencing and targeted liquid chromatography–tandem mass spectrometry, respectively. LPS challenge increased the relative abundance of *Fusobacteriota* and enriched several taxa associated with inflammatory dysbiosis, including *Proteobacteria*, *Enterobacteriaceae*, and *Escherichia-Shigella.* Compared with the LPS group, PAC supplementation was associated with a lower relative abundance of *Firmicutes* and higher relative abundances of *Mitsuokella* and *Dorea.* LPS challenge reduced the serum concentrations of several primary and secondary BAs and increased the concentrations of sulfated BAs. PAC supplementation was associated with lower concentrations of several BAs but a higher glycoursodeoxycholic acid (GUDCA) concentration than LPS alone. GUDCA was negatively correlated with several LPS-enriched taxa. These findings indicate that PAC supplementation was associated with changes in selected fecal bacterial taxa and serum BA concentrations during LPS challenge. Whether these microbial and BA changes are causally related requires further investigation.

## 1. Introduction

The weaning period is a critical stage in pig production, during which piglets are more susceptible to severe stress reactions because of their immature digestive and immune systems [1,2]. Studies have shown that weaning stress causes atrophy of intestinal villi, downregulation of tight junction protein expression, increased intestinal permeability, and disrupted gut microbiota homeostasis, thereby severely affecting the growth and health of piglets [3,4]. Microbiota dysbiosis further weakens intestinal barrier function, creating a vicious cycle that ultimately impairs the digestion and absorption of nutrients [5]. In weaned piglets, LPS challenge provides a practical model for examining how acute inflammatory stress affects intestinal function and host-associated microbial communities. Dietary interventions that help preserve intestinal homeostasis and limit stress-related disturbances in the gut microbiota are therefore important for supporting the health and growth of weaned piglets.

As the “key regulator” of gut health in piglets, the gut microbiota maintains intestinal homeostasis through various mechanisms. During the early stages of a piglet’s life, a stable gut microbiota contributes to the development of the host immune system, promotes the digestion and absorption of nutrients, and enhances the body’s resistance to disease. A healthy microbiota not only strengthens the intestinal epithelial barrier function and reduces inflammatory responses but also inhibits the colonization of pathogens through competitive exclusion and the secretion of antimicrobial substances [6,7]. Microbial products also participate in host signaling. For example, short-chain fatty acids can activate GPR43-related pathways, whereas tryptophan metabolites can influence mucosal immunity through the AHR-IL-22 axis [5,8]. Therefore, strategies regulating homeostasis of gut microbiota in weaned piglets are of great importance for improving intestinal health.

Beyond their established role in lipid digestion and absorption, bile acids (BAs) are increasingly recognized as signaling mediators involved in metabolic homeostasis, intestinal barrier maintenance, and immune regulation [9,10]. Previous studies indicate that individual BAs differ in their effects on intestinal physiology [11,12]. Chenodeoxycholic acid (CDCA), taurocholate, and ursodeoxycholic acid (UDCA) have been linked to barrier protection, immune regulation, and reduced intestinal inflammation, partly involving BA receptor signaling and microbiota-related pathways [13,14]. Intestinal microorganisms and BAs form a reciprocal metabolic relationship. Through deconjugation and subsequent biotransformation, microbial enzymes reshape the primary BA pool and generate secondary BAs. These microbial transformations alter the availability of BA receptor ligands and may consequently influence hepatic BA synthesis and enterohepatic homeostasis [15,16]. Furthermore, microbiota-dependent changes in the BA pool may also influence the host expression of BA synthesis enzymes, including CYP7A1, CYP27A1, and CYP7B1 [17]. Consequently, microbial remodeling of BAs can affect host metabolic and immune processes [18,19,20,21]. Conversely, BAs shape intestinal microbial communities through their detergent-like antimicrobial activity and receptor-mediated host responses [22,23,24,25]. Together, these reciprocal interactions provide a biological basis for the gut microbiota–BA axis.

Before restrictions were introduced, in-feed antibiotics were widely used to reduce health and performance losses associated with weaning in piglets [4]. With restrictions on antibiotic use in pig production, plant-derived bioactive components have attracted increasing attention as potential strategies for supporting intestinal health in weaned piglets. Proanthocyanidins (PACs), polyphenolic compounds widely found in plants, have been shown to effectively improve weaning stress in piglets, prevent intestinal inflammation, and thereby improve production performance [26,27]. PACs may also support epithelial barrier integrity, nutrient utilization, and digestive function, although these effects appear to depend on PAC source and dosage [28,29]. In the same experimental cohort, we previously reported that dietary supplementation with PACs improved growth performance, intestinal morphology, and barrier-related indices and altered ileal TGR5/GLP-2-related responses in LPS-challenged piglets [30]. Building on our previously reported findings, the present study investigated fecal microbial composition, serum BA profiles, and their associations to further characterize the responses associated with dietary PAC supplementation in LPS-challenged weaned piglets.

Accordingly, this exploratory secondary analysis evaluated whether dietary PAC supplementation was associated with changes in fecal microbiota composition and serum BA profiles in LPS-challenged weaned piglets. We hypothesized that PAC supplementation would be associated with selected microbial and BA changes and explored correlations between bacterial taxa and individual BAs. These correlations were interpreted as hypothesis-generating associations rather than evidence of a causal microbiota–BA mechanism.

## 2. Materials and Methods

### 2.1. Materials

Proanthocyanidins (PACs, HPLC ≥ 95%) were obtained from Tianjin Jianfeng Natural Products Research and Development Co., Ltd. (Tianjin, China). An LPS preparation isolated from Escherichia coli O55:B5 (L2880; Sigma-Aldrich, St. Louis, MO, USA) was used to induce inflammatory stress and was sourced from Sigma-Aldrich (catalog no. L2880, St. Louis, MO, USA).

### 2.2. Animals and Grouping

All animal procedures were authorized by the Institutional Animal Care and Use Committee of the Guangdong Laboratory Animals Monitoring Institute under protocol IACUC2024101. Eighteen 21-day-old Duroc × Landrace crossbred barrows, with a mean initial body weight (BW) of 7.16 ± 1.66 kg, were sourced from Conghua Danan Agricultural Development Co., Ltd. (Guangzhou, China). Only barrows were included; therefore, the findings cannot be directly generalized to gilts or intact males. Following a 3-day adaptation period, the animals entered a 21-day feeding trial conducted at the Guangdong Laboratory Animals Monitoring Institute. The experimental framework and host physiological outcomes from this cohort have been reported previously [30]. The present study analyzes distinct and previously unpublished fecal 16S rRNA gene sequencing and quantitative serum BA data obtained from the same animals. Piglets were stratified according to initial BW and randomly allocated to the CON, LPS, and PAC treatments, with six animals in each group. The CON group was fed the basal diet and administered saline intraperitoneally. Animals in the LPS group consumed the basal diet and received an intraperitoneal LPS challenge at 100 μg/kg BW. The PAC group was provided with the basal diet containing 250 mg/kg PACs and subjected to the same LPS regimen. The supplementation level was selected according to previous evidence [31]. LPS was freshly dissolved in sterile 0.9% saline. The administered dose was adjusted according to the individual BW of each piglet. Piglets in the LPS and LPS + PAC groups received an intraperitoneal injection of LPS at 100 μg/kg BW on days 14 and 21, whereas piglets in the CON group received an equivalent volume of sterile saline. Blood was obtained 2 h after the final injection, followed by anesthesia, euthanasia, and biological sample collection.

### 2.3. Experimental Management

Each piglet was maintained in a separate pen and had unrestricted access to the experimental diet and drinking water. The animal room was controlled at 28 °C and 45–55% relative humidity under a 12-h light/12-h dark schedule. Feed was replenished at 08:00, 12:00, 16:00, and 20:00 each day.

### 2.4. Diet Formulation

The diets used in the experiment were formulated in accordance with the NRC (2012) nutritional requirements for pigs and based on the net energy system. The ingredient formulation and nutrient composition of the experimental diet are presented in Table S1. No antibiotics were added to any of the experimental diets.

### 2.5. Sample Collection and Processing

On day 21, piglets in the LPS and PAC groups received an intraperitoneal injection of LPS under fasting conditions, whereas piglets in the CON group received an equal volume of saline. Two hours after injection, blood samples were collected into vacuum tubes and allowed to clot at room temperature for 30 min. The samples were centrifuged at 3500 rpm for 10 min at 4 °C. Serum was collected, divided into aliquots, and stored at −80 °C until bile acid profiling.

Following blood collection, the piglets were anesthetized and euthanized. Fecal samples were collected from the rectum into sterile cryovials, immediately frozen in liquid nitrogen, and stored at −80 °C until 16S rRNA gene sequencing.

### 2.6. 16S rRNA Gene Sequencing and Sequence Processing

Total microbial DNA was isolated from fecal samples with the E.Z.N.A. Stool DNA Kit (Omega Bio-tek, Norcross, GA, USA) following the supplier’s protocol. The bacterial V4 region was amplified with sample-indexed primers 515F (5′-GTGCCAGCMGCCGCGGTAA-3′) and 806R (5′-GGACTACHVGGGTWTCTAAT-3′). Each 15 μL PCR mixture comprised Phusion High-Fidelity PCR Master Mix (New England Biolabs, Ipswich, MA, USA), both primers at 0.2 μM, and approximately 10 ng of DNA template. Thermal cycling began with denaturation at 98 °C for 1 min, followed by 30 cycles at 98 °C for 10 s, 50 °C for 30 s, and 72 °C for 30 s, and ended with a 5-min extension at 72 °C.

Following magnetic-bead purification, the amplicons were quantified and combined at equal molar concentrations. Library concentration and quality were evaluated by Qubit fluorometry, quantitative PCR, and bioanalyzer analysis. Paired-end sequencing (2 × 250 bp) was subsequently conducted on an Illumina NovaSeq platform.

Raw paired-end reads were demultiplexed according to sample-specific barcodes, after which the barcode and primer sequences were removed. Paired-end reads were merged using FLASH v1.2.11, and the resulting raw tags were quality-filtered using fastp v0.23.1 to obtain clean tags. Chimeric sequences were identified by comparison with the SILVA 138.1 reference database and removed using VSEARCH v2.16.0. The resulting effective tags were denoised using the DADA2 plugin implemented in QIIME 2 v2022.2 to generate amplicon sequence variants (ASVs), and taxonomic identities were assigned against SILVA 138.1. For downstream diversity analyses, the ASV table was rarefied to 58,465 sequences per sample.

### 2.7. Targeted Analysis of Serum Bile Acids

Targeted bile acid quantification was conducted by MetWare Biotechnology Co., Ltd. (Wuhan, China). For metabolite extraction, a 50 μL serum aliquot was combined with 10 μL of internal standard solution (1 μg/mL) and 200 μL of 20% methanol–acetonitrile. Samples were mixed at 2500 rpm for 10 min and chilled at −20 °C for 10 min. Following centrifugation at 12,000 rpm and 4 °C for 10 min, the liquid phase was transferred and dried. The resulting extract was redissolved in 100 μL of aqueous 50% methanol for instrumental analysis.

Chromatographic analysis employed an ExionLC AD UPLC system coupled to an ACQUITY UPLC HSS T3 C18 column (100 × 2.1 mm, 1.8 μm; Waters, Milford, MA, USA). Eluent A comprised water supplemented with 0.01% acetic acid and 5 mmol/L ammonium acetate, whereas eluent B was acetonitrile containing 0.01% acetic acid. The column was maintained at 40 °C, with a flow rate of 0.35 mL/min and an injection volume of 3 μL. The A:B gradient was programmed as follows: 95:5 at 0 min, 60:40 at 0.5 min, 50:50 at 4.5 min, 25:75 at 7.5 min, 5:95 at 10 min, and 95:5 at 12 min.

An electrospray ionization-equipped QTRAP 6500+ system (SCIEX, Framingham, MA, USA) was used for mass spectrometric detection in negative-ion mode. The ion source was operated at 550 °C with a spray voltage of −4500 V and a curtain-gas pressure of 35 psi. Analytes were monitored by multiple reaction monitoring, using transition-specific declustering potentials and collision energies.

### 2.8. Statistical Analysis

Each piglet was regarded as an independent experimental unit. Statistical analyses were performed using R version 4.6.1 within RStudio 2026.07.0+139 (Posit Software, PBC, Boston, MA, USA), and bar charts were prepared using GraphPad Prism 9.5. Data are presented as mean ± SEM. The normality of the residuals from the one-way ANOVA models was assessed using the Shapiro–Wilk test. Alpha-diversity indices, relative abundances of dominant taxa, and serum bile acid concentrations were analyzed by one-way ANOVA. When the overall treatment effect was significant, Fisher’s least significant difference (LSD) test was applied for pairwise comparisons. For the results presented in tables, means without a common superscript letter were considered significantly different at *p* < 0.05. Statistical significance was defined as *p <* 0.05, whereas 0.05 ≤ *p* < 0.10 was considered indicative of a statistical tendency. Principal coordinate analysis based on unweighted UniFrac distances was used to visualize differences in fecal microbial community structure among groups, whereas statistical differences were assessed using pairwise PERMANOVA based on Bray–Curtis dissimilarity matrices. Differentially abundant taxa among groups were identified using linear discriminant analysis effect size (LEfSe), with a Kruskal–Wallis test threshold of *p* < 0.05 and an LDA score > 2.0. Associations between bacterial taxa and serum bile acids were evaluated using Spearman’s rank correlation. A nominal *p*-value < 0.05 was used as the threshold for the exploratory correlation analysis.

## 3. Results

### 3.1. Effects of PAC Supplementation on the Fecal Microbiota of Weaned Piglets

#### 3.1.1. Shared and Unique ASVs

**Figure 1:**
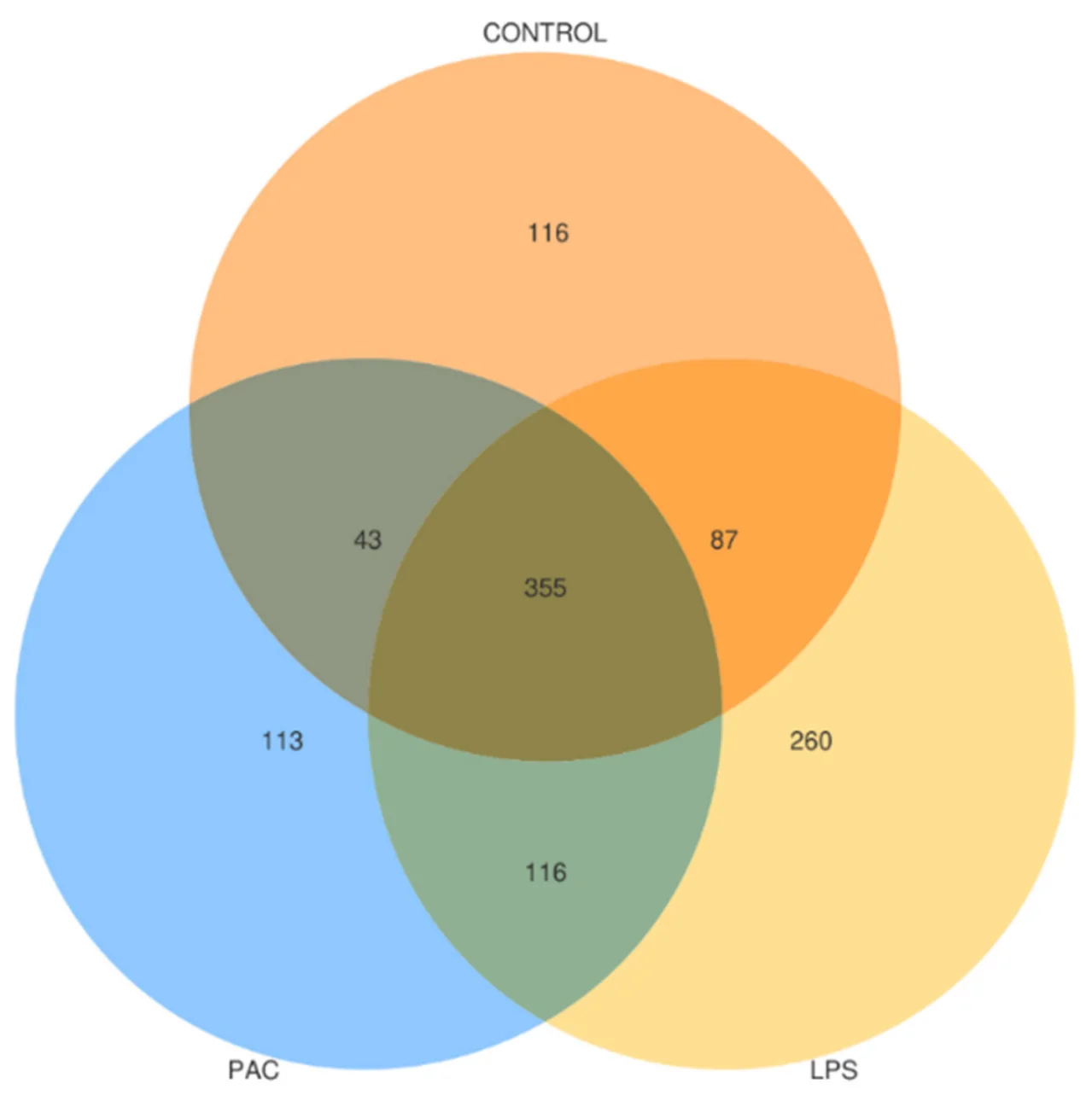
Venn diagram showing shared and unique bacterial amplicon sequence variants (ASVs) among the CON, LPS, and PAC groups.

The distribution of shared and unique Amplicon Sequence Variants (ASVs) among the three groups is shown in Figure 1. A total of 1090 ASVs were identified across the three groups, of which 355 were shared by all three groups, accounting for 32.57% of the total. The numbers of ASVs unique to the CON, LPS, and PAC groups were 116, 260, and 113, respectively. In addition, 87, 43, and 116 ASVs were shared exclusively between the CON and LPS, CON and PAC, and LPS and PAC groups, respectively.

#### 3.1.2. Alpha Diversity

**Figure 2:**
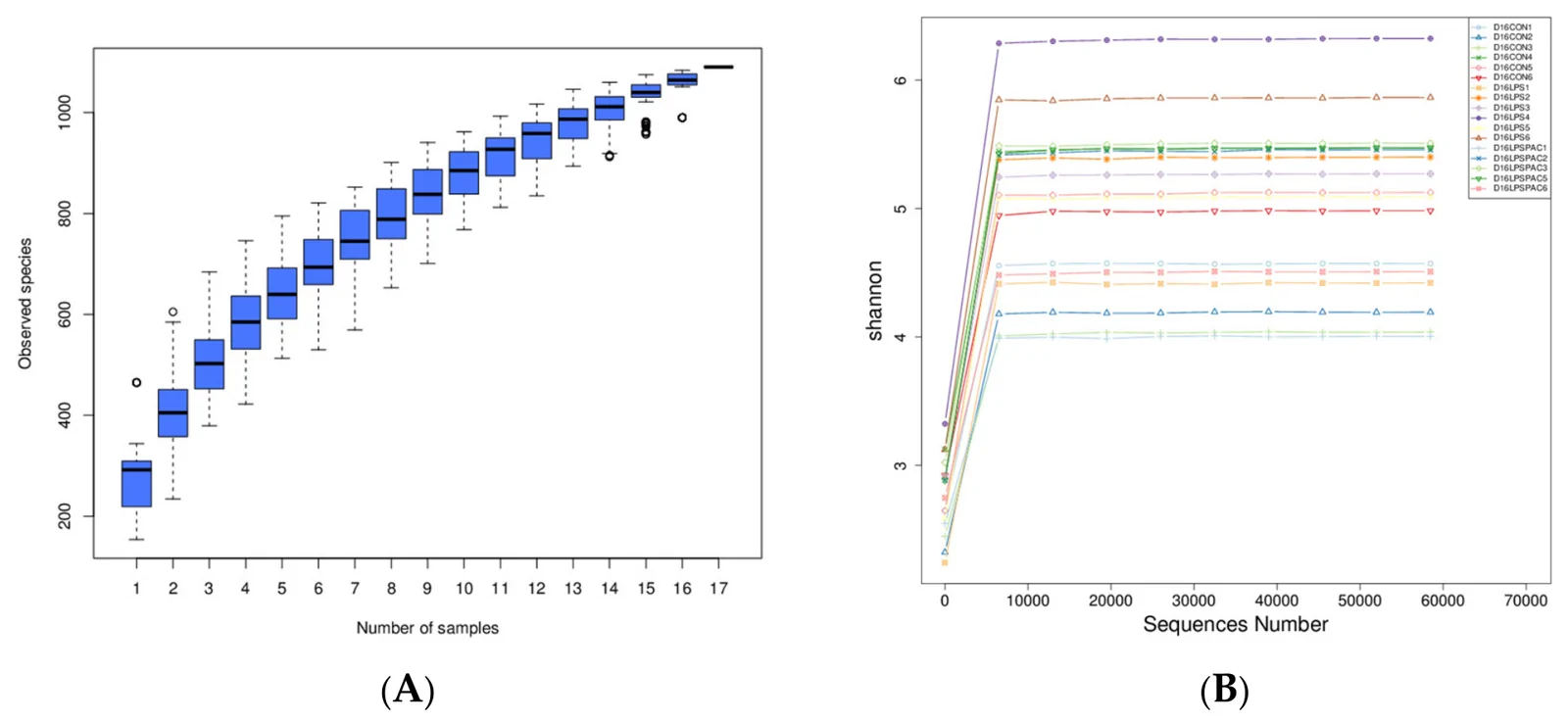
Rarefaction curves of observed amplicon sequence variants (ASVs) and Shannon diversity in fecal bacterial communities (cutoff = 58,465). (**A**) Rarefaction curves; (**B**) Shannon curves.

Rarefaction curves based on observed ASVs and Shannon diversity approached plateaus for all samples, indicating that the sequencing depth was sufficient for downstream diversity analysis (Figure 2). No significant differences were observed in any alpha-diversity index among the three groups (*p* > 0.05; Table 1).

#### 3.1.3. Beta Diversity

**Figure 3:**
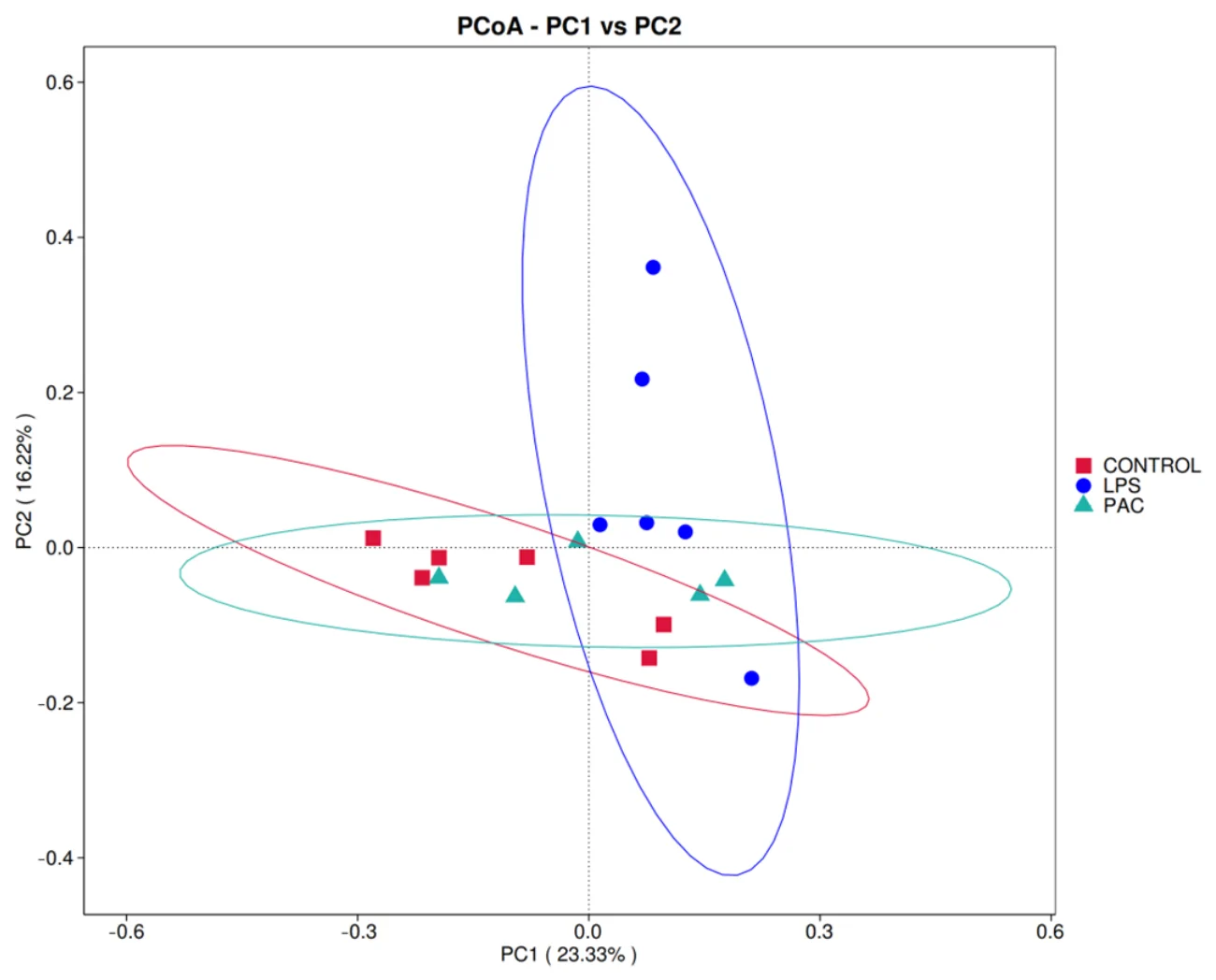
Principal coordinates analysis (PCoA) based on unweighted UniFrac distances.

Principal coordinate analysis based on unweighted UniFrac distances showed visual separation among the CON, LPS, and LPS + PAC groups (Figure 3). Pairwise PERMANOVA based on Bray–Curtis dissimilarities indicated a tendency toward a difference in microbial community structure between the CON and LPS groups after Benjamini–Hochberg correction (pseudo-F = 2.011, R^2^ = 0.167, q = 0.072). No significant differences were detected between the CON and LPS + PAC groups (pseudo-F = 0.833, R^2^ = 0.085, q = 0.566) or between the LPS and LPS + PAC groups (pseudo-F = 1.129, R^2^ = 0.111, q = 0.455).

#### 3.1.4. Phylum and Genus Microbiota Level Differences

**Figure 4:**
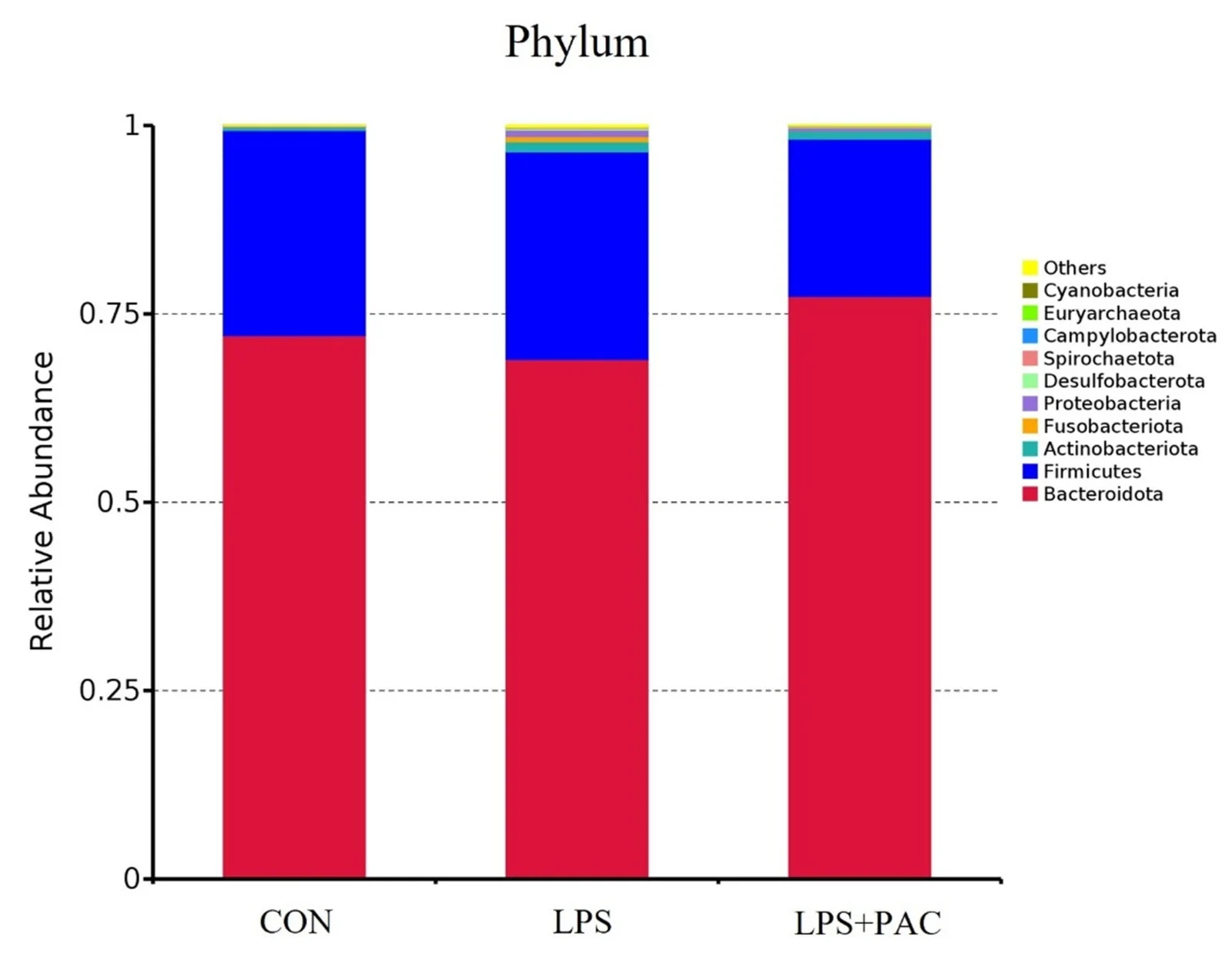
Relative abundances of bacterial phyla in fecal samples from the CON, LPS, and PAC groups.

Bacteroidota and Firmicutes were the dominant phyla in the fecal bacterial communities (Figure 4). The relative abundance of Fusobacteriota was higher in the LPS group than in the CON group (*p* < 0.05). Compared with the LPS group, PAC supplementation reduced the relative abundance of Firmicutes (*p* < 0.05; Table 2). Proteobacteria showed a tendency to differ among the three groups (*p* = 0.099).

**Figure 5:**
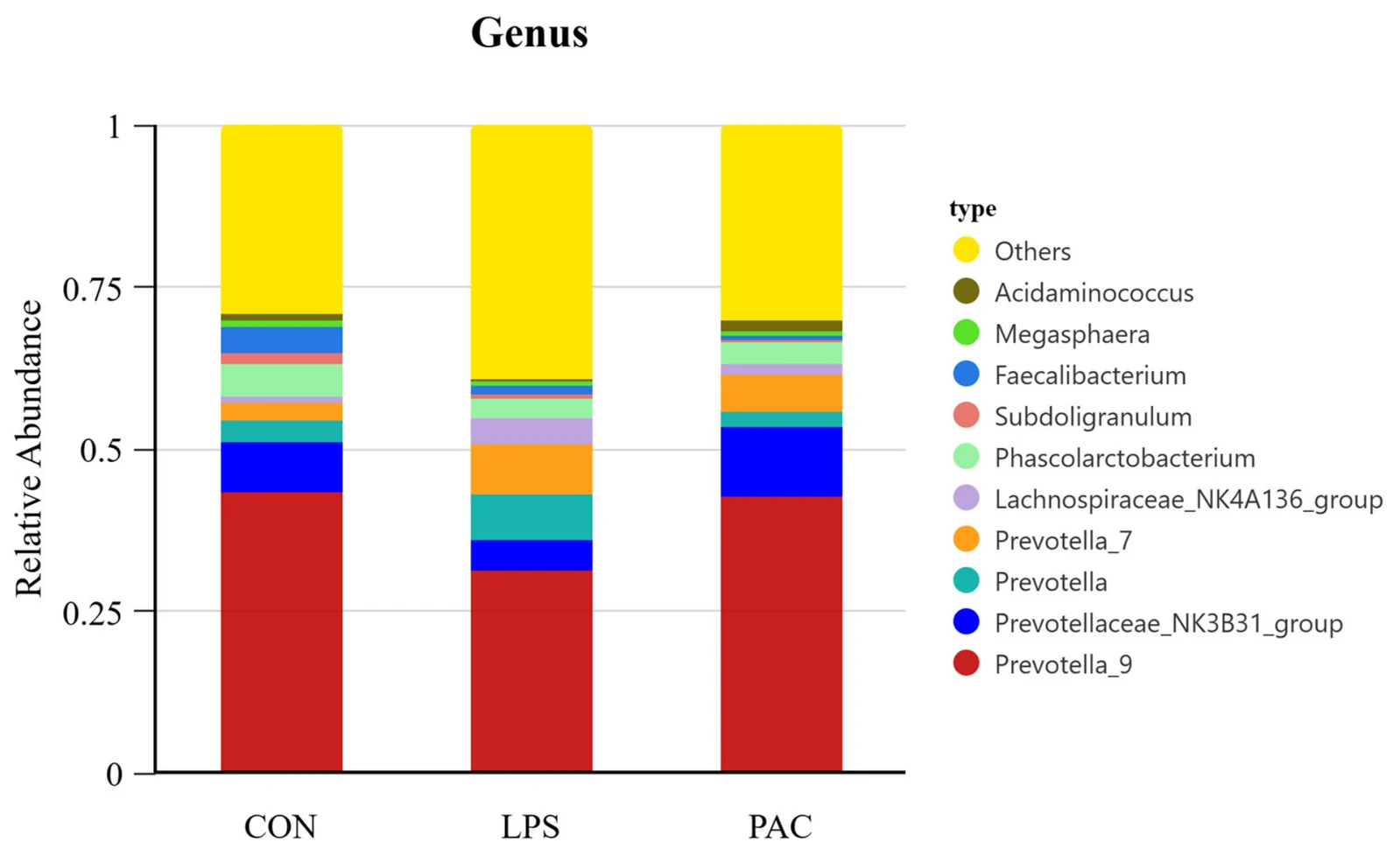
Relative abundances of bacterial genera in fecal samples from the CON, LPS, and PAC groups.

The most abundant genera included *Prevotella_9*, *Prevotellaceae_NK3B31_group*, Prevotella, *Prevotella_7*, *Lachnospiraceae_NK4A136_group*, *Phascolarctobacterium*, *Subdoligranulum*, *Faecalibacterium*, *Megasphaera*, and *Acidaminococcus* (Figure 5). Compared with CON, the LPS group had lower *Prevotella_9* and *Acidaminococcus* and higher *Prevotella_7*, *Alloprevotella*, and *[Eubacterium]_eligens_group* (*p* < 0.05). Compared with LPS, the PAC group had higher *Acidaminococcus* and *Collinsella* and lower *Prevotella_7*, *Alloprevotella*, and *[Eubacterium]_eligens_group* (*p* < 0.05; Table 2).

#### 3.1.5. LEfSe Analysis

**Figure 6:**
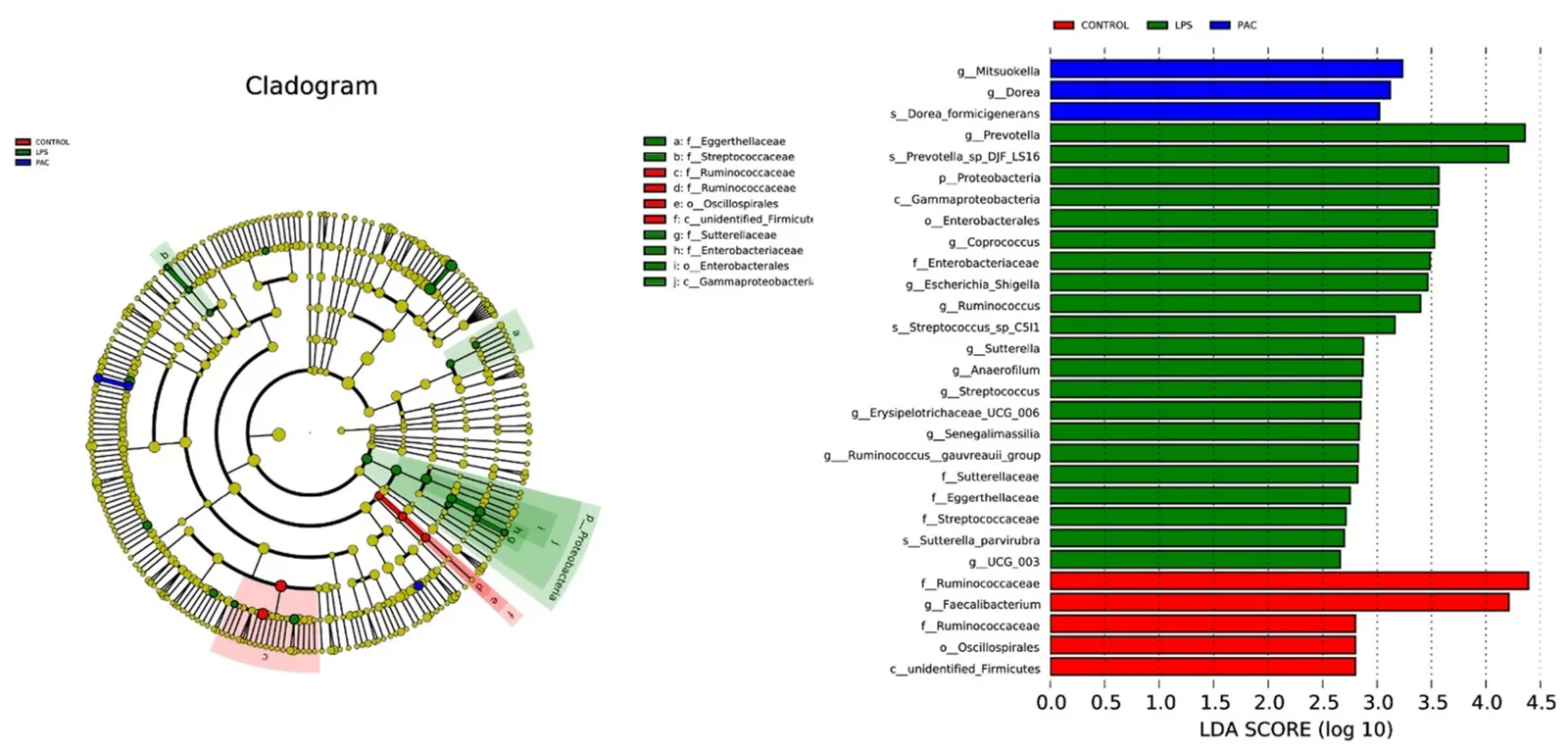
LEfSe analysis of differential bacterial taxa among groups. (**Left**) Taxonomic cladogram. (**Right**) Linear discriminant analysis scores.

To identify microbial taxa that showed significant differences between groups, a Linear discriminant analysis Effect Size (LEfSe) analysis was performed, and the results are shown in Figure 6. The analysis revealed that the Ruminococcaceae family, *Faecalibacterium* genus, Oscillospirales order, and unidentified Firmicutes were significantly enriched in the CON group, whereas in the LPS group, the *Prevotella genus*, *Prevotella_sp_DJF_LS16*, *Proteobacteria*, *Enterobacterales*, *Coprococcus*, *Enterobacteriaceae*, *Escherichia-Shigella*, *Ruminococcus*, and *Streptococcus_sp_C5i1*, *Sutterella*, *Anaerofilum*, *Streptococcus*, *Erysipelotrichaceae_UCG_006*, *Senegalimassilia*, *Ruminococcus gauvreauii group*, *Sutterellaceae*, *Eggerthellaceae*, *Sutterella parvirubra*, *UCG_003*, and other opportunistic pathogens or potentially harmful bacteria were significantly enriched. In contrast, the groups significantly enriched in the PAC group were *Mitsuokella* and *Dorea*, both of which are closely associated with carbohydrate fermentation metabolism.

### 3.2. Effects of PAC Supplementation on Serum BA Profiles

Hyocholic acid (HCA), glycohyocholic acid (GHCA), chenodeoxycholic acid (CDCA), and hyodeoxycholic acid (HDCA) were among the most abundant serum BAs (Table 3). LPS challenge reduced taurohyocholic acid (THCA), CDCA, glycochenodeoxycholic acid (GCDCA), taurochenodeoxycholic acid (TCDCA), glycohyodeoxycholic acid (GHDCA), and glycoursodeoxycholic acid (GUDCA), while increasing BA sulfate (*p* < 0.05). Compared with LPS, PAC supplementation further reduced THCA, CDCA, GCDCA, and GHDCA but increased GUDCA (*p* < 0.05). Taurohyodeoxycholic acid showed a statistical tendency across groups (*p* = 0.070).

### 3.3. Correlation Analysis Between Gut Microbiota and Serum BAs

**Figure 7:**
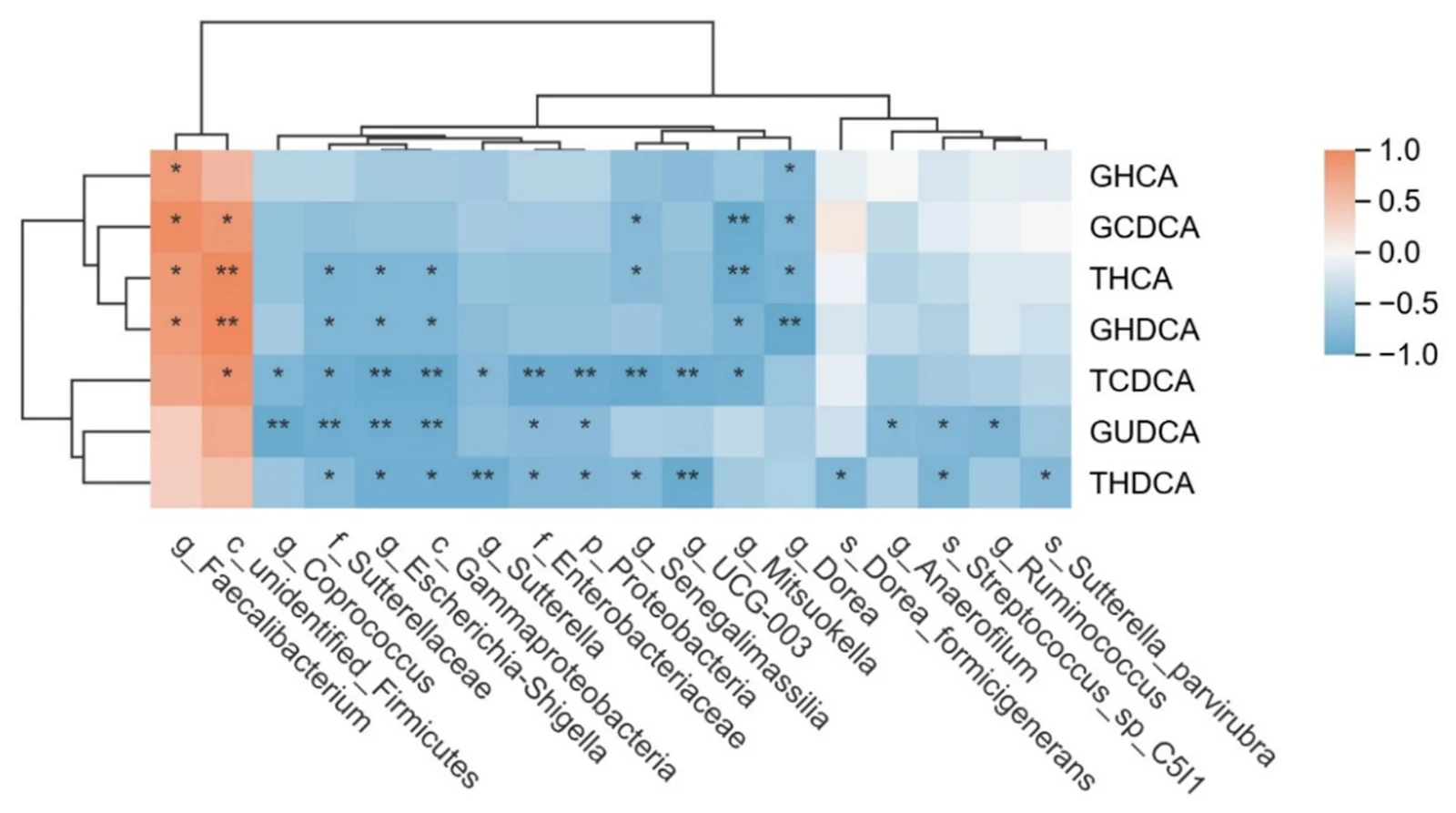
Spearman correlations between differential bacterial taxa and serum BAs. Note: Blue indicates a negative correlation, and orange indicates a positive correlation. * *p* < 0.05; ** *p* < 0.01. Note: Glycohyocholic acid (GHCA), Glycochenodeoxycholic acid (GCDCA), Taurohyocholic acid (THCA), Glycohyodeoxycholic acid (GHDCA), Taurochenodeoxycholic acid (TCDCA), Glycoursodeoxycholic acid (GUDCA), Taurohyodeoxycholic acid (THDCA).

Spearman correlation analysis was performed between LEfSe-identified taxa and BAs that differed among groups (Figure 7). At the nominal *p* < 0.05 threshold, GUDCA was negatively associated with several LPS-enriched taxa, including Escherichia–Shigella, Enterobacteriaceae, and Proteobacteria. In contrast, Faecalibacterium and unidentified Firmicutes were positively associated with several primary and secondary BAs.

## 4. Discussion

### 4.1. Effects of PAC on the Gut Microbiota of Weaned Piglets

The gut microbiota plays a crucial role in various aspects of piglet nutrition, metabolism, immune regulation, and intestinal development [32,33]. During the weaning transition, abrupt dietary and environmental changes can disturb gut microbial homeostasis in piglets, and the resulting dysbiosis has been associated with impaired intestinal barrier function and immune regulation, potentially increasing susceptibility to enteric pathogens [34]. This study found that although there were no significant differences in α-diversity indices among groups, the relative abundance of gut microbiota underwent significant changes at the phylum and genus levels, consistent with previous research findings [35]. LPS challenge significantly increased the relative abundance of *Fusobacteriota*. *Fusobacteriota* is a Gram-negative anaerobic pro-inflammatory bacterial phylum, whose enrichment impairs the intestinal barrier and exacerbates the inflammatory response. LEfSe further identified *Proteobacteria*, *Enterobacteriaceae, Escherichia-Shigella, Sutterella*, and *Streptococcus* as taxa enriched in the LPS group. *Proteobacteria*, *Enterobacteriaceae* and *Escherichia-Shigella* are typical opportunistic pro-inflammatory taxa, suggesting a shift toward a microbial community associated with inflammatory stress. These results suggest that LPS challenge was associated with alterations in fecal microbial composition in piglets, consistent with previous studies [36]. In contrast, the addition of PACs to the diet significantly reduced the abundance of the *Firmicutes* phylum and enriched the genera *Mitsuokella* and *Dorea*. Both genera have been associated with carbohydrate fermentation capabilities, and their metabolic products (such as acetic acid and butyric acid) play a positive role in intestinal immune homeostasis and barrier integrity [37]. Previous in vitro studies have shown that PACs may exert differential effects on bacterial taxa by altering the membrane integrity of Staphylococcus aureus and inhibiting the growth and biofilm formation of Escherichia coli, while exerting weaker inhibitory effects on beneficial bacteria such as Lactobacillus [38]. Previous studies have linked PAC-induced microbial changes to intestinal short-chain fatty acids (SCFAs) production and immune regulation [39,40]. Consistent with this possibility, PAC supplementation in the present study was associated with reduced abundance of several LPS-enriched taxa and enrichment of *Mitsuokella* and *Dorea*. However, because microbial metabolites were not directly measured here, the functional implications of these taxa-level changes remain to be verified. Furthermore, because the microbiota analysis was based on fecal samples, these findings should not be extrapolated directly to site-specific microbial communities in other gastrointestinal compartments.

### 4.2. Effects of PAC on BA Metabolism in Weaned Piglets

BA homeostasis is critical for maintaining intestinal health in piglets [41]. Previous research by our group found that high levels of secondary BAs can disrupt intestinal tight junction proteins, thereby damaging the intestinal barrier [42]. BAs and BA receptors play important regulatory roles in animal intestinal physiology [43], and their involvement in the regulation of immune cells has been well supported by previous studies [44,45,46]. In the present study, HDCA and HCA were among the most abundant serum BAs, consistent with previous reports in pigs [47]. LPS stimulation altered BA profiles in piglets, manifested by significantly reduced levels of various primary BAs (THCA, CDCA, GCDCA) and secondary BAs (GHDCA, GUDCA) in serum, accompanied by elevated levels of sulfated BAs. BA sulfation, which is mediated predominantly by hepatic sulfotransferases such as SULT2A1, increases BA hydrophilicity and facilitates the elimination of potentially cytotoxic BAs through urine or feces [48]. Although sulfated BAs represented only a small fraction of the serum BA pool in the present study, their increase may reflect an adaptive response to altered BA homeostasis following LPS challenge. Similar alterations in BA sulfation have been reported under cholestatic and inflammatory conditions [49,50]. However, the physiological relevance of this quantitatively minor change remains to be determined. The results of this study indicate that while PACs intervention further reduced the levels of some primary BAs, it significantly increased GUDCA, which has shown anti-inflammatory effects in other experimental models. Prachi Agnihotri et al. [51] found that GUDCA can reduce levels of inflammatory factors in SW982 cells and increase IGF-1 expression. Huang et al. (2022) found in a neurodegenerative model that GUDCA can reduce TNF-α and IL-6 levels [52]. These observations suggest that the increase in GUDCA may be one BA-related feature associated with the response to PACs, although its direct contribution to intestinal inflammation was not tested in this study. Previous work has reported that GUDCA can act as a mediator linking gut microbiota changes with FXR signaling, partly through interactions involving Bacteroides fragilis [53]. In the present study, GUDCA was negatively correlated with several LPS-enriched taxa, including Enterobacteriaceae, Escherichia-Shigella, Sutterella, and Streptococcus. Some members of these taxa, including Enterobacteriaceae, may participate in BA transformation through bile salt hydrolase-related activity [54]. These associations suggest a possible link between PAC-related microbial shifts and serum GUDCA changes. Together, these findings suggest a possible association between PAC-related microbial shifts and changes in serum BA profiles, with GUDCA emerging as a potentially relevant BA. Similar microbiota-BA links have been proposed in other dietary intervention studies, such as the reported regulation of host BA metabolism by quercetin through Akkermansia muciniphila [55]. In the same experimental cohort, we previously reported that dietary PAC supplementation improved intestinal morphology and barrier-related indices and altered ileal TGR5/GLP-2-related responses under LPS challenge [30]. The changes in selected fecal bacterial taxa and serum BA concentrations observed in the present study provide complementary microbial and metabolic context for these previously reported host responses. However, the present associations do not establish that the microbial changes caused the serum BA responses or mediated the intestinal effects of PACs.

Serum and fecal BA profiles provide distinct and complementary information. Serum BAs reflect the absorbed and circulating BA pool after intestinal uptake, hepatic modification, and enterohepatic recirculation, whereas fecal BAs more directly represent the unabsorbed and excreted luminal BA pool. Therefore, the serum BA concentrations measured in the present study cannot be used to infer the relative production of individual BAs in the intestine. Concurrent profiling of serum and fecal BAs will be required to distinguish intestinal microbial BA transformation from systemic BA metabolism.

The use of a sex-homogeneous cohort of barrows reduced sex-related biological variation, but further studies are needed to determine whether these findings also apply to gilts and intact males. In addition, the use of a highly purified PAC preparation (HPLC ≥ 95%) may constrain commercial feasibility. Future studies should evaluate sex-related responses, commercially relevant feed-grade PAC preparations, dose optimization, feed-processing stability, and cost–benefit relationships.

## 5. Conclusions

In LPS-challenged weaned piglets, dietary supplementation with 250 mg/kg PAC was associated with selected changes in fecal microbial composition and serum BA concentrations. Relative to the LPS group, PAC supplementation was associated with a lower relative abundance of Firmicutes, higher relative abundances of Mitsuokella and Dorea, and a higher serum GUDCA concentration, although several other BA concentrations were lower. The observed associations between GUDCA and selected LPS-enriched taxa do not establish a causal microbiota–BA relationship. Further studies with larger sample sizes are needed to confirm these findings.

## Figures and Tables

**Table 1:** Alpha-diversity indices of fecal bacterial communities in weaned piglets.

Items	Treatments			*p*-Value
	CON	LPS	LPS + PAC	
chao1	221.41 ± 10.12	314.05 ± 10.99	264.33 ± 12.80	0.108
dominance	0.09 ± 0.01	0.08 ± 0.01	0.08 ± 0.01	0.770
observed_features	221.17 ± 10.18	313.17 ± 11.02	263.60 ± 12.65	0.109
pielou_e	0.61 ± 0.01	0.65 ± 0.01	0.62 ± 0.01	0.116
shannon	4.71 ± 0.08	5.39 ± 0.10	4.99 ± 0.12	0.222
simpson	0.91 ± 0.01	0.92 ± 0.01	0.92 ± 0.01	0.770

Note: Data are expressed as mean ± SEM. Means without a common superscript letter differ significantly (*p* < 0.05).

**Table 2:** Effects of dietary PAC supplementation on the relative abundances of fecal bacterial taxa in weaned piglets.

Items	Treatments			*p*-Value
	CON	LPS	PAC	
Phylum				
*Firmicutes*	24.374 ± 1.99 ^a^	25.425 ± 1.183 ^a^	20.891 ± 1.997 ^b^	0.015
*Bacteroidota*	75.116 ± 1.999	71.115 ± 1.338	77.390 ± 2.127	0.296
*Actinobacteriota*	0.3913 ± 0.029	1.047 ± 0.227	1.051 ± 0.138	0.626
*Fusobacteriota*	0.000 ± 0.001 ^b^	0.781 ± 0.192 ^a^	0.028 ± 0.010 ^ab^	0.034
*Proteobacteria*	0.001 ± 0.000	1.094 ± 0.184	0.361 ± 0.078	0.099
*Desulfobacterota*	0.001 ± 0.001	0.203 ± 0.052	0.071 ± 0.019	0.316
*Spirochaetota*	0.103 ± 0.031	0.088 ± 0.014	0.180 ± 0.045	0.165
*Campylobacterota*	0.001 ± 0.001	0.020 ± 0.008	0.002 ± 0.001	0.961
*Euryarchaeota*	0.004 ± 0.002	0.002 ± 0.001	0.019 ± 0.004	0.557
*Cyanobacteria*	0.003 ± 0.001	0.003 ± 0.001	0.005 ± 0.002	0.730
*Firmicutes*/*Bacteroidota* ratio	0.411 ± 0.097	0.417 ± 0.069	0.299 ± 0.107	0.618
Genus				
*Prevotella_9*	49.390 ± 1.503 ^a^	34.109 ± 2.275 ^b^	42.782 ± 3.175 ^ab^	0.031
*Prevotellaceae_NK3B31_group*	3.424 ± 0.685 ^b^	4.547 ± 0.6826 ^b^	10.672 ± 2.319 ^a^	0.002
*Prevotella*	3.891 ± 0.557 ^a^	6.462 ± 0.897 ^ab^	2.341 ± 0.160 ^b^	0.045
*Phascolarctobacterium*	5.713 ± 0.559	2.728 ± 0.381	3.586 ± 0.483	0.072
*Faecalibacterium*	4.276 ± 0.411	0.975 ± 0.146	0.663 ± 0.140	0.118
*Prevotella_7*	3.132 ± 0.413 ^b^	6.897 ± 0.592 ^a^	5.673 ± 0.978 ^b^	0.011
*Lachnospiraceae_NK4A136_group*	0.692 ± 0.062	4.529 ± 0.743	1.541 ± 0.221	0.305
*Subdoligranulum*	5.125 ± 0.478 ^a^	0.571 ± 0.133 ^b^	3.586 ± 0.483 ^ab^	0.015
*Acidaminococcus*	0.931 ± 0.247 ^a^	0.198 ± 0.029 ^b^	0.315 ± 0.043 ^a^	0.008
*Alloprevotella*	0.744 ± 0.066 ^b^	1.039 ± 0.114 ^a^	0.663 ± 0.140 ^c^	0.026
*Parabacteroides*	0.320 ± 0.038	0.787 ± 0.120	0.578 ± 0.141	0.053
*unidentified_Prevotellaceae*	1.350 ± 0.357	0.363 ± 0.065	1.565 ± 0.384	0.231
*Agathobacter*	1.652 ± 0.310 ^a^	0.381 ± 0.091 ^ab^	0.213 ± 0.076 ^b^	0.030
*Blautia*	1.841 ± 0.206 ^a^	1.403 ± 0.092 ^ab^	1.239 ± 0.218 ^b^	0.041
*Megamonas*	1.393 ± 0.495	0.691 ± 0.151	0.578 ± 0.141	0.276
*Bacteroides*	0.108 ± 0.020	0.606 ± 0.145	0.741 ± 0.220	0.173
*UCG-002*	0.073 ± 0.015	0.970 ± 0.197	0.427 ± 0.071	0.098
*[Eubacterium]_eligens_group*	0.686 ± 0.144 ^b^	1.000 ± 0.126 ^a^	0.805 ± 0.128 ^b^	0.014
*unidentified_Oscillospiraceae*	0.063 ± 0.006	0.142 ± 0.021	0.119 ± 0.020	0.345
*Fusobacterium*	0.001 ± 0.000	0.510 ± 0.191	0.0281 ± 0.010	0.248
*Collinsella*	0.264 ± 0.024 ^b^	0.562 ± 0.153 ^ab^	0.742 ± 0.122 ^a^	0.018
*Rikenellaceae_RC9_gut_group*	0.319 ± 0.047	0.545 ± 0.070	0.765 ± 0.144	0.065
*Holdemanella*	0.208 ± 0.036	0.304 ± 0.047	0.044 ± 0.005	0.153
*Escherichia-Shigella*	0.036 ± 0.014	0.639 ± 0.156	0.300 ± 0.073	0.105

Note: Data were log-transformed before analysis and are presented as the mean ± SEM. Means without a common superscript letter differ significantly (*p* < 0.05).

**Table 3:** Effects of dietary PAC supplementation on serum bile acid profiles in weaned piglets.

Items	Treatments			*p*-Value
	CON	LPS	LPS + PAC	
Primary Bile Acids (ng/mL)				
Hyocholic acid (HCA)	6134.45 ± 306.31	4031.78 ± 303.16	4717.79 ± 530.30	0.561
Glycohyocholic acid (GHCA)	1290.37 ± 172.78	521.77 ± 20.74	441.28 ± 62.77	0.082
Taurohyocholic acid (THCA)	266.27 ± 15.49 ^a^	61.31 ± 2.28 ^b^	48.95 ± 7.66 ^c^	<0.01
Chenodeoxycholic acid (CDCA)	1240.81 ± 131.31 ^a^	726.03 ± 22.29 ^b^	369.91 ± 32.85 ^c^	<0.01
Glycochenodeoxycholic acid (GCDCA)	151.26 ± 1.78 ^a^	69.01 ± 1.95 ^b^	34.32 ± 3.53 ^c^	<0.01
Taurochenodeoxycholic acid (TCDCA)	29.49 ± 1.43 ^a^	10.45 ± 0.18 ^b^	10.52 ± 0.57 ^b^	<0.01
Cholic acid (CA)	1.63 ± 0.06	2.15 ± 0.21	0.68 ± 0.31	0.522
Glycocholic acid (GCA)	14.48 ± 0.36 ^a^	0.00 ± 0.00 ^b^	0.00 ± 0.00 ^b^	<0.01
Taurocholic acid (TCA)	0.00 ± 0.00	0.00 ± 0.00	0.00 ± 0.00	1.000
Secondary Bile Acids (ng/mL)				
Hyodeoxycholic acid (HDCA)	1754.79 ± 305.39	2409.17 ± 122.46	2234.46 ± 548.09	0.868
Glycohyodeoxycholic acid (GHDCA)	795.79 ± 43.85 ^a^	261.34 ± 12.71 ^b^	204.61 ± 36.72 ^c^	0.005
Taurohyodeoxycholic acid (THDCA)	124.49 ± 18.51	17.89 ± 1.41	62.03 ± 9.35	0.070
Ursodeoxycholic acid (UDCA)	0.00 ± 0.00	0.00 ± 0.00	0.00 ± 0.00	1.000
Glycoursodeoxycholic acid (GUDCA)	653.12 ± 42.03 ^a^	208.02 ± 10.21 ^c^	323.31 ± 51.65 ^b^	0.030
Tauroursodeoxycholic acid (TUDCA)	66.84 ± 10.23	13.62 ± 1.23	49.95 ± 7.59	0.138
Lithocholic acid (LCA)	1.74 ± 0.25	1.50 ± 0.01	4.86 ± 1.12	0.249
Glycolithocholic acid (GLCA)	0.77 ± 0.25	0.00 ± 0.00	1.19 ± 0.44	0.436
Taurolithocholic acid (TLCA)	0.17 ± 0.04	0.31 ± 0.05	0.00 ± 0.00	0.533
Deoxycholic acid (DCA)	0.20 ± 0.04	0.30 ± 0.00	0.33 ± 0.11	0.389
Glucuronide	1.891 ± 0.22	1.927 ± 0.50	0.532 ± 0.25	0.372
Bile acid sulfate (BA-Sulfate)	3.51 ± 0.12 ^b^	6.47 ± 0.08 ^a^	5.86 ± 0.01 ^a^	0.003

Note: Data are expressed as mean ± SEM. Means without a common superscript letter differ significantly (*p* < 0.05).

## Data Availability

The original data presented in the study are openly available in NCBI SRA at PRJNA1482325.

## Supplementary Materials

The following supporting information can be downloaded at: https://www.mdpi.com/article/10.3390/microorganisms14102324/s1, Table S1: Composition and nutrient levels of the basal diets (air-dry basis).

## Abbreviations

The following abbreviations are used in this manuscript:

PACs	Proanthocyanidins
LPS	Lipopolysaccharide
LC-MS/MS	Liquid Chromatography–Tandem Mass Spectrometry
THCA	Taurohyocholic acid
CDCA	Chenodeoxycholic acid
GCDCA	Glycochenodeoxycholic acid
GHDCA	Glycohyodeoxycholic acid
GUDCA	Glycoursodeoxycholic acid
BA-Sulfate	Bile acid sulfate
BW	Body weight
HCA	Hyocholic acid
GHCA	Glycohyocholic acid
TCDCA	Taurochenodeoxycholic acid
CA	Cholic acid
GCA	Glycocholic acid
TCA	Taurocholic acid
HDCA	Hyodeoxycholic acid
THDCA	Taurohyodeoxycholic acid
UDCA	Ursodeoxycholic acid
TUDCA	Tauroursodeoxycholic acid
LCA	Lithocholic acid
GLCA	Glycolithocholic acid
TLCA	Taurolithocholic acid
DCA	Deoxycholic acid
SCFAs	Short-chain fatty acids
ASVs	Amplicon Sequence Variants
LEfSe	Linear discriminant analysis Effect Size
