# Supplementary material for: Dietary Proanthocyanidins Are Associated with Changes in the Fecal Microbiota and Serum Bile Acid Profiles of LPS-Challenged Weaned Piglets
Source: Microorganisms. 2026 Oct 12;14(10):2324. doi: 10.3390/microorganisms14102324 (PMC13707881; doi:10.3390/microorganisms14102324)
Supplement: Supplementary file 1 [file microorganisms-14-02324-s001.zip › microorganisms-4540385-supplementary.pdf]

Table S1 Composition and nutrient levels of the basal diets (air-dry basis)

Ingredients	Content%	Nutrition level[3]	Content%
Corn	28.85	Digestible energy, MJ/kg	14.83
Expanded corn	10.00	Metabolizable energy, MJ/kg	14.23
Extruded soybean	14.00	Net energy, MJ/kg	10.25
Fermented soybean meal	11.50	Crude protein %	21.11
Soybean powder	7.00	Calcium Ca%	0.90
Fishmeal	3.00	Total phosphorus%	0.63
Whey powder	15.00	SID Lys%	1.40
Whey protein concentrate	1.00	SID Met%	0.40
Soyabean oil	1.00	SID Thr%	0.82
Sucrose	3.00	SID Trp%	0.23
Calcium citrate	1.85	SID Ile%	0.72
Dicalcium phosphate	0.60		
Sodium chloride	0.25		
L-Lysine	0.55		
DL-Methionine	0.15		
L-Threonine	0.20		
L-Tryptophan	0.05		
L- Valine	0.10		

Choline chloride	0.20
Vitamin premix[1]	0.40
Mineral premix[2]	1.30

[1]The vitamin premix Provided the following per kg of the diet: Vitamin A, 12000 IU; Vitamin B_1, 5 mg; Vitamin B_2, 6 mg; Niacin, 20 mg; Pantothenic acid, 20 mg; Vitamin B_6, 3 mg; Biotin, 360 μg; Folic acid, 2 mg; Vitamin B_{12}, 30 μg; Vitamin D_3, 5000 IU; Vitamin E, 50 mg; Vitamin K_3, 5 mg;

[2]The mineral premix Provided the following per kg of the diet: Fe($FeSO_4 \cdot H_2O$), 100 mg; Zn($ZnSO_4 \cdot H_2O$), 100 mg; Cu($CuSO_4 \cdot 5H_2O$), 6 mg; Mn($MnSO_4 \cdot H_2O$), 4 mg; Se(Na_2SeO_3), 0.5 mg; I(KI), 0.2 mg.

[3] Nutritional levels are calculated.
